# Current Indications for Surgical Repair in Patients with Bicuspid Aortic Valve and Ascending Aortic Ectasia

**DOI:** 10.1155/2012/313879

**Published:** 2012-09-20

**Authors:** Christian D. Etz, Martin Misfeld, Michael A. Borger, Maximilian Luehr, Elfriede Strotdrees, Friedrich-Wilhelm Mohr

**Affiliations:** ^1^Department of Cardiac Surgery, Leipzig Heart Center, University of Leipzig, Strümpellstraße 39, 04289 Leipzig, Germany; ^2^Department of Cardiothoracic Surgery, Mount Sinai School of Medicine, New York, NY 10029, USA

## Abstract

Preventive surgical repair of the moderately dilated ascending aorta/aortic root in patients with bicuspid aortic valve (BAV) is controversial. Most international reference centers are currently proposing a proactive approach for BAV patients with a maximum ascending aortic/root diameter of 45 mm since the risk of dissection/rupture raises significantly with an aneurysm diameter >50 mm. Current guidelines of the European Society of Cardiology (ESC) and the joint guidelines of the American College of Cardiology (ACC)/American Heart Association (AHA) recommend elective repair in symptomatic patients with dysfunctional BAV (aortic diameter ≥45 mm). In asymptomatic patients with a well-functioning BAV, elective repair is recommended for diameters ≥50 mm, or if the aneurysm is rapidly progressing (rate of 5 mm/year), or in case of a strong family history of dissection/rupture/sudden death, or with planned pregnancy. As diameter is likely not the most reliable predictor of rupture and dissection and the majority of BAV patients may never experience an aortic catastrophe at small diameters, an overly aggressive approach almost certainly will put some patients with BAV unnecessarily at risk of operative and early mortality. This paper discusses the indications for preventive, elective repair of the aortic root, and ascending aorta in patients with a BAV and a moderately dilated—or ectatic—ascending aorta.

## 1. Introduction: Brief History and ****Epidemiology 

Probably the first to ever visualize a bicuspid aortic valve was Leonardo da Vinci during his studies on the geometric characteristics of the human aortic valve 500 years ago—as precisely documented by his drawings. William Osler—one of the pioneers of modern medicine—was the first to recognise the clinical relevance of the bicuspid geometry of the aortic valve in 1886. Dr. Paget had described the liability of bicuspid aortic valves to valve disease even earlier, in 1844, before Peacock in 1858 recognised that BAVs have a particular tendency to develop stenosis and regurgitation. Since the late 20th century BAV has been known as the most prevalent congenital heart defect with an incidence of 1-2% [1]. 

In Germany, approximately 800,000 to 1,600,000 patients are born with a bicuspid aortic valve and the majority is likely to develop valve and/or ascending aortic/root complications by the age of 70 [2, 3]. In only 20% of these patients the bicuspid aortic valve remains competent for a lifetime. The incidence of patients with bicuspid aortic valve among patients requiring aortic valve surgery is approximately 30% [4, 5].

As opposed to their normal peers with a tricuspid aortic valve, patients with BAV develop more severe valve pathologies more rapidly, often times with the risk of progressive congestive heart failure, and exhibit a significantly higher incidence of pathological changes of the ascending aorta/aortic root [2, 6–13]. 

## 2. Ascending Aorta/Aortic Root: Normal Aorta, Ectasia, or Aneurysm?

The normal diameter of the ascending aorta may be influenced by gender and BMI but seems to independently be associated with patient age [14]. 

Hannuksela et al. used radiographic data from a normal population to develop a formula that allows for calculation of upper normal diameter of the ascending aorta (*D*) in relation to patient age [14]:

(1) $$\begin{array}{c} D\;\left(\text{mm}\right)=31+0.16\times \text{age}\;\left(\text{years}\right). \end{array}$$

A modification is added in case of extreme weight:

(2) $$\begin{array}{c} D\;\left(\text{mm}\right)=21+0.14\times \text{age}\;\left(\text{years}\right)+\left(0.41\times \text{BMI}\right). \end{array}$$

Accordingly, an ascending aortic diameter of 34 mm is still “normal” in a 20-year-old patient (average diameter 27 mm). In an 80-year-old patient (average diameter 37 mm) a diameter of 44 mm is still classified “normal”.

According to the classic understanding, a diameter increase of 50% marks the borderline between ectasia and aneurysm—the threshold at which a dilated ascending aorta/root should be considered an aneurysm, therefore, is

 
**~**40** **mm in a 20-year-old
 
**~ **45** **mm in a 40-year-old
 
**~ **50** **mm in a 60-year-old
 
**~ **55** **mm in an 80-year old

norm-weighed patient, and according to Hannuksela et al.'s norm diameters [14].

These benchmarks for normal, age-related diameters, however, cannot reliably guarantee freedom from aortic complications, particularly in patients with BAV.

Although it still remains unclear whether mechanical characteristics of the aortic wall are related to size or body mass, some groups have developed a variety of “biometric indices” to allow for risk stratification.

## 3. Indices Used to Risk Stratify Patients with a Dilated Thoracic Aorta

In 2006, Davies et al. proposed an aortic size index for risk stratification and surgical indication in patients with thoracic aortic aneurysms [15]. 

Svensson et al. at the Center of Aortic Surgery of the Cleveland Clinic implemented a ratio to calculate operative risk in 2003, by using the following formula including aortic width (*r*), cross-section area, and patient height:

(3) $$\begin{array}{c} \frac{r^{2}\times \pi \;\left(\text{c}{\text{m}}^{2}\right)}{\text{height}\;\left(\text{m}\right)} \end{array}$$

## 4. BAV—Associated Aortic Pathology

Bicuspid aortic valve has been increasingly recognised as a pathology of the entire proximal ascending aorta, including the aortic annulus, the sinus of valsalva, the coronary ostia, the sinutubular junction, and the tubular part of the ascending aorta [16, 17]. 

The involvement of the transverse, and even the distal arch beyond the ligamentum arteriosum, is currently controversial and affects—if at all—only a small minority of BAV patients [18, 19]. Today, most clinicians agree that the distal arch and the descending and thoracoabdominal aorta are not generally involved in the pathology of bicuspid aortic valve [18, 20]. Interestingly, the proximal pulmonary artery appears to also be dilated in a significant number of patients with BAV, possibly due to the same embryonic derivation from neural crest cells. This knowledge might increasingly influence surgical strategies, particularly in the preventive therapy of younger BAV patients that yet had been considered excellent candidates for the Ross procedure [21, 22]. 

### 4.1. Clinical Relevance of BAV—Associated Proximal Aortic Pathology

In BAV patients, aortic root and ascending aortic aneurysms appear to occur more frequently and more importantly at a younger age than in normal controls with tricuspid aortic valves (TAVs) [8, 12]. Approximately 4 out of 10 patients develop a dilation of the ascending aorta of more than ≥40 mm [23]. In comparison with TAV patients with a similar aortic diameter, the aortic wall in patients with BAV appears to be thinner with decreased distensibility, resulting in a higher risk for rupture and acute dissection that increases with diameter [10, 24–27]. 

Clinically, BAV must be considered a disease of the entire proximal aorta: the root and the tubular ascending aorta [28, 29]. The most relevant vascular complications comprise rapid aneurysmatic dilation and acute type A aortic dissection [2, 13, 30].

### 4.2. Prevalence of Aortic Ectasia in Patients with BAV

**Figure 1:**
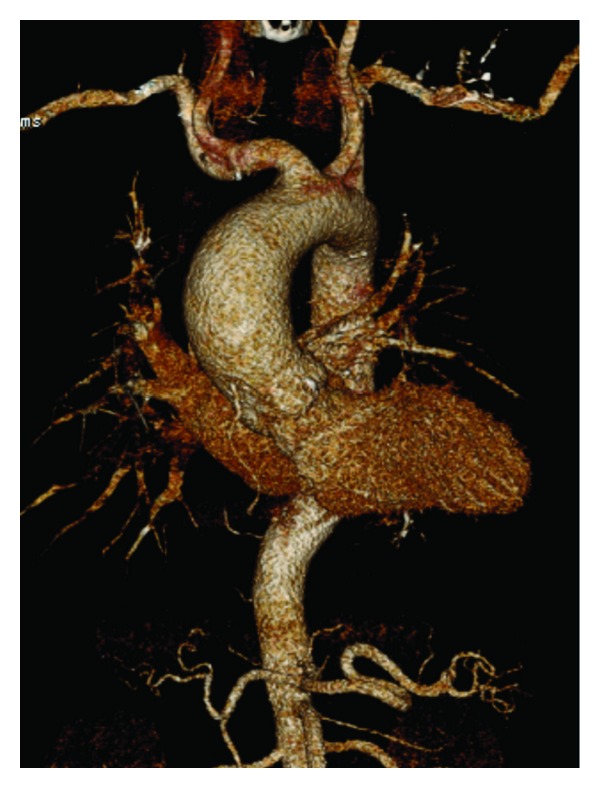
Moderately dilated ascending aorta of a young BAV patient. 3D reconstruction (CT angiography) of a typical ascending aortic aneurysm of a young BAV patient.

**Figure 2:**
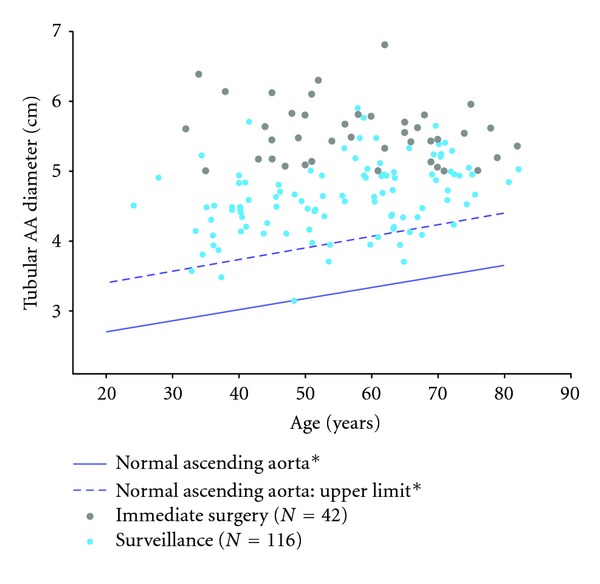
From Etz et al. [8]. Tubular AA diameter at index computed tomographic scan versus age of each individual patient entering the program; patients under surveillance (*n* = 116) versus immediate surgery (*n* = 42). (Data for normal ascending aorta (∗) and normal ascending aorta: upper limit (∗) derived from Hannuksela et al. [14].

Ectasia of the central ascending aorta without or with only marginal involvement of the aortic root seems to be the most frequent variant of aortic involvement (Figure 1). However, not only extent but also the exact location of the aortic ectasia appears to be distributed heterogeneously over the BAV population, as is the risk of aortic complications (Figure 2) [31–33].

Interestingly, the proportion of male : female BAV patients with a surgical indication for ascending aortic replacement is almost uniformly described with 4 : 1, reflecting the gender distribution of BAV in the normal population [34]. 

It is known, that the normal ascending/root diameter is associated with age [14]. Several studies focusing on the aortic involvement in BAV patients reported on a relatively early aortic dilation with a progressive course affecting 88% of patients at age 80 [2, 17, 35, 36]. 

**Figure 3:**
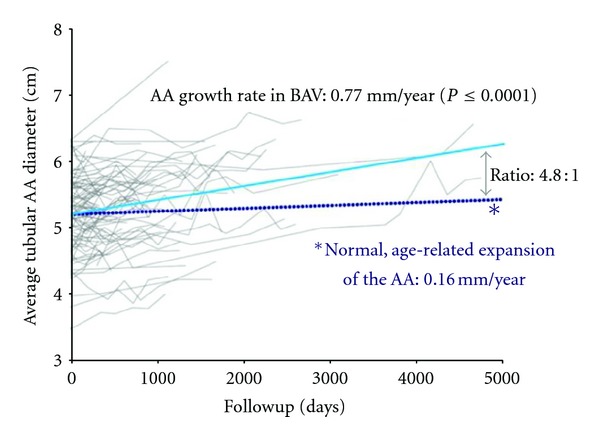
From Etz et al. [8]. Average growth of the ascending aorta in patients (*n* = 116) with normally functioning bicuspid aortic valve versus normal, age-related expansion. (Data for dotted line in this figure are derived from Hannuksela et al. [14].

Comparing the ascending diameters in patients with BAV and a normal population, the expansion rate of the ascending aorta appears significantly higher in patients with BAV, even in the presence of a normal, nondysfunctional BAV (Figure 3) [8]. Della Corte and colleagues analysed 280 patients with a nondysfunctional BAV and determined the growth rate with <1 mm/year [31].

Longitudinal studies revealing long-term, population-based data and conclusive evidence are scarce, conclusions controversial. In 2008, Michelena and colleagues reported on a longitudinally followed cohort of 212 initially asymptomatic patients (age, 32 ± 20 years; 65% male, all community residents from Olmsted County, MN, USA), echocardiographically diagnosed with nondysfunctional bicuspid aortic valves: ascending aorta dilatation (>40 mm) was noted in 15% at baseline and in 39% at followup [23]. In this study, during a follow-up period of 20 years, 8 patients required surgery for ascending aorta dilatation or aneurysm, leading to a 20-year rate of 5 ± 2% [23]. Overall, aortic valve surgery, ascending aortic surgery, or any cardiovascular surgery was required at a younger age than in the age- and sex-matched general population (*P* < 0.0001) [23]. Survival 20 years after diagnosis, however, was 90 ± 3%, identical to an age- and sex-matched general population (*P* = 0.72) [23]. Interestingly, in this study no aortic dissection had occurred amongst the 212 patients with nondysfunctional BAV during the 20-year followup [23].

In 2011, in a subsequent study on 416 consecutive patients with BAV (regardless of valve function) from the Olmsted County population, published in JAMA by the same authors found the incidence of aortic dissection over a mean of 16 years of followup to be low but significantly higher than in the general population [32]: aortic dissection occurred in 2 male patients, type A in one and type B in the other (valve status was postaortic valve replacement in one and moderate aortic stenosis in the other), resulting in an incidence of 3.1 (95% CI, 0.5–9.5) cases per 10,000 patient-years, or an age-adjusted relative risk of 8.4 (95% CI, 2.1–33.5; *P* = 0.003) compared with the county's general population [32]. The two patients that dissected had 46 mm and 47 mm measurements at baseline with their respective last measurements before dissection as 52 mm and 50 mm (unfortunately, the authors do not report on the interval between the last measurement and the time the dissection occurred). 

Whether patients with BAV do dissect at a younger age or a smaller diameter than their tricuspid peers is yet unclear. *Valve function clearly has an impact on aneurysm progression.*

### 4.3. Progression of Proximal Aortic Dilation and Valve Function

Aortic stenosis was the only multivariate valve-related predictor of ascending aneurysm formation in patients with BAV, associated with an hazard ratio (95% CI) of 3.4 (1.8–6.3; *P* < 0.001) in this recent population-based, retrospective cohort study by Michelena and colleagues from Mayo Clinic, published in JAMA in September 2011 [32]. A baseline aorta diameter >40 mm was the only other significant multivariate predictor with a hazard ratio of 3.3 (1.5–7.2; *P* < 0.004), in other words: once a BAV patient reaches an ascending diameter of 40 mm, the risk of aneurysm formation requiring open surgery is significantly increased [32].

The association between ascending aortic/root dilation and functional valve status remains controversial [2, 18, 33, 37]. 

### 4.4. Poststenotic Dilatation Affects the Midascending Aorta

Della Corte and colleagues in their study on 280 adult patients with isolated BAV found severe aortic stenosis to be one of only two (age between 50–60 years being the second with an odds ratio of 13.7; reference category: <30 years) independent predictors of dilatation of the tubular, mid-ascending aorta with an odds ratio of 23.8 (*P* < 0.001) and observed a positive correlation between the degree of stenosis and the actual midascending diameter (*P* = 0.016, when excluding small aortas from the analysis) [31].

Accordingly, Ben-Dor and colleagues found a significant (*P* < 0.001) enlargement of the annulus (24.1 ± 2.8 versus 21.4±1.8 mm) and the ascending aorta (39 ± 6.9 versus 31.3 ± 3.7 mm) diameters in patients with stenotic BAV versus normal, tricuspid controls, with ascending aortic dimensions above the upper normal range (37 mm) in 60% of the bicuspid group (*P* < 0.001); however, no difference between stenotic BAV and controls with regard to the diameter of the sinuses and the sinotubular junction was observed [38]. 

### 4.5. Root Dilatation and Regurgitant BAV

Severe aortic regurgitation was with an odds ratio of 3.9 (*P* = 0.011), one of three determinants of root involvement (the others were again age >60 with an odds ratio of 2.6, *P* = 0.022 and male gender with an odds ratio of 4.1, *P* = 0.001); interestingly, aortic valve stenosis was a protective factor for root dilatation (odds ratio 0.3, *P* < 0.001) [31].

Roberts and colleagues recently in an unadjusted comparison among 96 patients with congenitally bicuspid aortic valves found significant differences in the loss of elastic fibers in the media of the resected ascending aorta (aortic wall tissue of the root/sinuses was not explicitly included!): patients with purely regurgitant BAV had a much greater likelihood of significant aortic medial elastic fiber loss than those with stenotic BAV (unadjusted OR: 8.8; 95% CI: 2.95, 28.13) [33]. Compared to normal controls with tricuspid valve, patients with purely regurgitant BAV were 35 times more likely (unadjusted OR: 35.2; 95% CI: 1.98, 624.57) to have significant loss of medial elastic fibers, while no significant differences in elastic fiber loss were observed between patients with stenotic BAV and the control subjects (unadjusted OR: 4.0; 95% CI: 0.21, 75.9) [33]. Amongst the BAV patients, those with a regurgitant valve had a significantly higher likelihood of significant elastic fiber loss than those with a stenotic valve (crude odds ratio: 8.8; 95% confidence interval: 2.95, 28.13) [33]. The major and surprising finding of this study was that 90% of the patients with a stenotic valve had no or only a minimal loss of medial elastic fibers and only 10% had a significant loss. The authors concluded that “*these findings support the view that patients with aortic stenosis and an aneurysmally dilated ascending aorta infrequently need to have the aorta replaced with a graft*” [33].

Roberts et al.'s study might renew the discussion in three major aspects: (1) as to whether a mild to moderately dilated proximal aorta in a patient with nondysfunctional BAV really requires early, preventive surgery, (2) if patients undergoing aortic valve replacement for stenotic valve disease do really benefit from a “proactive” approach to ascending replacement or can be treated more conservatively, and, last not least (3) if patients with aortic regurgitation should undergo ascending/root repair earlier and/or more radically. Since neither aortic root tissue nor tissue of the distal ascending aorta/proximal arch was explicitly included and analyzed in this study, more specific questions on the most beneficial extent of the repair (e.g., which BAV patient needs root repair rather than supracommissural replacement, or which patients might benefit from additional hemiarch repair) are not going to be affected by this study. Furthermore, the dynamics of these remodeling processes—and possible clinical implications—are not yet clear.

Clinical studies comparing the long-term outcome of patients with stenotic BAV versus patients with a regurgitant BAV, however, did not reveal any significant differences between both groups [7].

Specific morphological aspects of the type of bicuspid valve—as proposed by Sievers and colleagues—have not been addressed by Roberts and colleagues in this report and since the functional status and the pattern of aortic dilation might be distinct phenomena of one mutual genetic origin, therapeutic consequences are to be drawn cautiously.

### 4.6. Morphology of the Bicuspid Aortic Valve and Prevalence of Aortic Ectasia

An association of cusp configuration and pattern of aortic dilation have been proposed by several surgical classification systems, based on intraoperative findings: a fusion of the left- and right-coronary cusp is most prevalent. Interestingly, this type appears to most infrequently be associated with root/ascending aortic ectasia. 

**Figure 4:**
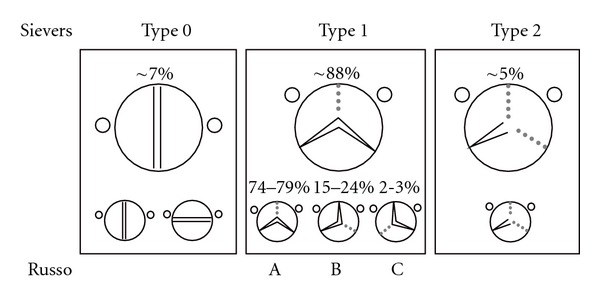
Shaded area shows the position of the raphe on the valve. LC-NC, Left-coronary-noncoronary cusp; LC-RC, left-coronary-right-coronary cusp; RC-NC, right-coronary-noncoronary cusp. Figure assembled according *to* Sievers and Schmidtke [41], and Russo et al. [39].

In 2008, Russo and colleagues introduced their classification of bicuspid aortic valves distinguishing the most frequent fusion of the left- and right-coronary cusp as type A (74%), fusion of the right- and noncoronary cups as type B (24%), and a rare fusion of left, and noncoronary cusp as type C (2%; see Figure 4). 

At the time of surgery, type A patients, despite of a comparable mean diameter of the ascending aorta of 49 mm (*P* = 0.34), had a significantly more dilated root (45 mm versus 33 mm; *P* < 0.0001) and were significantly younger than type B patients (51 versus 59 years, *P* = 0.034) [39]. Interestingly, there were no significant differences with regard to valve dysfunction in both groups [39, 40]. 

Sievers and colleagues proposed a systematic and precise classification system distinguishing three main categories according to the number of raphes which are further subcategorized by the position of the raphe (Figure 4): type 0 (true bicuspid), type 1, and 2. In their initial report, true bicuspid valves (type 0; 7%) and bicuspid valves with one raphe (type 1; 88%; with a fusion of the left and right cusp (LR), the right, and noncoronary cusp (RN), or the noncoronary and left (NL) cusp) accounted for 95% of patients, however, were associated only in ~10% with an ectasia of the root and only in ~25% with an aneurysm of the ascending aorta >50 mm. 

While only about 5% of BAV patients presented with the rare type 2 (fusion of two raphes), the type 2 patients significantly more often (*P* = 0.022) had an associated ascending aneurysm: in more than 60% of cases [41]. 

In Sievers et al.'s study population, however, ~95% of patients and in Russo et al.'s analysis 100% of patients had a dysfunctional bicuspid aortic valve, reducing the validity of risk stratification by cusp configuration.

## 5. An Association between BAV Cusp ****Morphology and the Risk of Type A Aortic ****Dissection Has Not Been Described Yet

### 5.1. Acute Type A Aortic Dissection in Patients with BAV

Aortic dissection can occur at any diameter and, particularly, at a smaller size than generally perceived. The risk of rupture and acute dissection appears to increase significantly once a diameter of 50 mm is reached [42, 43]. In 2003 Svensson and colleagues from Cleveland Clinic Foundation reported on a series of 430 patients undergoing surgical intervention for bicuspid aortic valves and ascending aorta with or without aortic arch repair at two major US hospitals; 40 patients had aortic dissection (of which 25 dissections were acute): 12.5% (*N* = 5) of all patients with dissection dissected at a maximum diameter of less than 50 mm [10, 43, 44]. Since about 15% of patients with Marfan's syndrome dissect their aorta at a size of less than 5 cm, the risk for patients with BAV appears comparable, but 1-2% of the population has a bicuspid valve [43, 44]. 

Autopsy studies had revealed that 9–15% of all patients with aortic dissection had a BAV [13, 45, 46]. In 1984 Larson and Edwards, in a necropsy study of 161 cases, claimed a 9-fold increased risk of aortic dissection for patients with BAV [46]. Acute aortic dissection might occur at a younger age in patients with BAV: data from the IRAAD (The International Register for Acute Aortic Dissection) suggested that patients under the age of 40 who suffered type A aortic dissection more often had a BAV than those dissecting over the age of 40 (9% versus 1%, *P* < 0.01) [36]—our own institutional data on more than 330 acute type A aortic dissections support these findings.

Svensson and colleagues found an increased dissection rate with a relationship of aortic cross-sectional size to height exceeding 10 cm^2^/m. Interestingly, dissection occurred at a smaller diameter for shorter patients [10].

Acute aortic dissection in patients with BAV may be associated with increased hospital mortality after emergency surgery and diminished longevity [7, 10, 13].

### 5.2. BAV and Ectasia of the Aorta in First-Degree Relatives

Biner and colleagues described the prevalence of aortic dilation in first-degree relatives of BAV patients, who themselves had a normal, tricuspid aortic valve: the aortic root was significantly wider in the patients with BAV than compared to their first-degree relatives. However, first-degree relatives had a significantly wider root than a normal control population with tricuspid valve who did not have relatives with BAV [47]. There were no differences with regard to the sinotubular junction between the three groups, and the tubular ascending aorta was dilated in patients with BAV only, not in their relatives, or the normal control [47]. Furthermore, Biner et al. found that the proximal aortic wall of patients with BAV and their first-degree relatives—independently of the diameter—was less distensable and significantly stiffer as compared to normal controls [47]. 

Maximum aortic diameter and diameter progression remain the most widely used criteria for preventive surgical repair, particularly in patients with suspected or confirmed connective tissue disease [15, 26, 42, 48]. 

### 5.3. Indication for Aortic Repair in Bicuspid Patients Undergoing Valvular Surgery

The current guidelines of the European Society of Cardiology (ESC) recommend concomitant repair for patients undergoing surgery for any degree of aortic regurgitation with a root diameter of more than 50 mm. However, in case of diameter progression of more than 5 mm per year or a positive family history of aortic dissection, the surgical indications of BAV patients are progressively converging towards the surgical indications in Marfan's syndrome (>45 mm of diameter). Moreover, the ESC guidelines suggest that in patients with an indication for aortic valve surgery even lower thresholds of aortic dilation (<45 mm) can be used for ascending aortic surgery. These surgical indications by the ESC also comprise BAV patients with significant valve stenosis [49]. Therefore, the European Association of Echocardiography (EAE) recommends serial echocardiograms in BAV patients with aortic root dilation (<50 mm) [50]. 

The conjoint guidelines of 2008 by the American College of Cardiology (ACC) and the American Heart Association (AHA) proposed a more aggressive approach in the presence of significant valve pathology, and suggest—as a class 1 recommendation—concomitant aortic root replacement in case of required valve reconstruction for severe aortic stenosis or regurgitation due to aortic root/ascending aortic diameter of >45 mm (level of evidence C) [51].

Therefore, aortic root/ascending aortic ectasia with a diameter of more than 45 mm is being treated concomitantly during aortic valve reconstruction/replacement of BAV patients in most aortic centers [7, 10, 18, 52]. 

Svensson et al. found an increased risk for aortic dissection in BAV patients with maximum aortic cross-section: body height ratio of 10 cm^2^/m and suggested prophylactic supracommissural replacement of the tubular ascending aorta in patients with symptomatic valve dysfunction and an aortic diameter of >45 mm *or a maximum aortic cross-section: body height ratio of *10 *cm* ^2^/*m* [10]. Recently, this strategy was confirmed in a large series of almost 2000 BAV patients in which Svensson et al. now even suggests a more aggressive, “proactive” approach towards supracommissural aortic replacement in patients with a maximum aortic diameter of >45 mm, *or a ratio of > *8-9 *cm* ^2^/*m*, or *z*-values of >7 (maximum aortic cross-section area/body hight). Interestingly, these operative criteria are also used for elective surgery on the proximal aorta in patients with Marfan's syndrome [52]. A more aggressive approach, in case of aortic diameters <45 mm, with regard to the current literature is not justifiably. In addition, Svennson et al. are convinced that extending surgery towards concomitant aortic root or hemiarch replacement is not indicated on an elective basis [52]. 

### 5.4. Operative Indications for BAV Patients without Valvular Pathology

Currently no expert consensus exists about the optimal time for elective surgery on the aortic root and the ascending aorta to prevent rupture or acute dissection in BAV patients with normal valvular function [7, 8]. The surgical indication for operative repair in patients with bicuspid valve and normal valve function is controversial due to the heterogeneity of BAV-associated complications and the associated degree of aortic dilation [31]. Some authors suggest to operate on patients with an aortic diameter of >55 mm, while others advocate for aortic root/ascending aortic replacement in BAV patients with an aortic diameter of under 45 mm. There is a current trend towards periodic routine followup and selective “proactive” surgery [8, 52]. 

The AHA/ACC guidelines recommend surgery with an aortic root or ascending aortic diameter of 50 mm confirmed via transesophageal echocardiography, independently from BAV function, *or* with dilation rate of ≥5 mm/year (level of evidence C) [51]. Moreover, the AHA/ACC guidelines point out that in aortic centers aortic root/ascending aortic reconstruction may be performed at an aortic diameter of 45 mm or a dilation rate of 5 mm/year (or more) [53]. In *asymptomatic* patients *without valvular pathology,* Svensson et al. suggest a ratio of >10 cm^2^/m (maximum aortic cross-section area/body height) for elective surgery [52].

The surgical indication for patients with a normally functioning BAV and a moderately dilated aorta should be made on an individual basis comprising valvular morphology and relevant comorbidities.

## 6. Current Recommendations for the Management of BAV Patients 

With regard to the current guidelines and literature we recommend the following approach for BAV patients with a moderately dilated aortic root/ascending aorta (and their family members).

1. Annual MRI (or CT angiography/aortic protocol) surveillance for all aortic diameters >40 mm or *any diameter above the age-related normal range [14] (e.g., in a 30-year-old patient starting with a maximum diameter of 36 mm and above): if rapid progression is suspected at an interval of 6 months* (with regard to diameter and progression), screening for first-degree family members, especially men: echocardiographic detection of valve morphology and aortic diameter.
2. BAV patients without valvular indication for surgery: elective aortic replacement if the following criteria for the aortic root/ascending aorta apply:
  - maximum diameter ≥50 mm, *or *
  - rapid growth progression of ≥0.5 cm/year, *or *
  - maximum aortic cross-sectional area/body height ≥10 cm^2^/m.

*Lower limits than 50 mm for maximum aortic diameter, a ratio of 10 cm* ^2^ */m (aortic cross-section/body height), or growth progression of 5 mm/year should be applied for patients with isolated aortic pathology, positive family history for aortic dissection/rupture, or unexplained sudden death in 1st degree relatives, as well as for female patients considering pregnancy. For patients with Sievers type 2 valve morphology we currently recommend a proactive approach, especially with beginning valve dysfunction. *

(3) BAV patient with valvular indication: concomitant aortic replacement if the following criteria for the aortic root/ascending aorta do apply:
  (a) maximum diameter ≥45 mm *or *
  (b)  maximum aortic cross-section area/body height ≥8-9 cm^2^/m.
(4) Annual transthoracic (if necessary transesophageal) echocardiography surveillance to evaluate valve function with immediate strategy revaluation in case of new occurring valve dysfunction.

In BAV patients with equivocal diameter criteria, the use of the aortic index and the ratio of maximum aortic cross-section area and body height is reasonable [10, 14]. In patients with a small body surface area it is reasonable to use an index of aortic diameter and BSA. 

Consideration of age-related norm values of aortic diameters is reasonable to plan for routine surveillance intervals [14].

An aggressive approach for aortic replacement in patients with clearly identified Sievers type 2 morphology is indicated even without existence of valve dysfunction. This may especially apply in the presence of other known risk factors (e.g., arterial hypertension) for aortic catastrophes or known familial disposition. 

In female BAV patients proximal aortic dilation can be induced by or progress to ectasia or aneurysm during pregnancy, while the risk of aortic dissection may be increased [54]. Therefore, we recommend routine surveillance during pregnancy, particular in patients with BAV, although there is no evidence. 

Optimal oral antihypertensive therapy during surveillance in patients with a borderline operative indication is obligatory and should comprise an “anti-impulse therapy” to reduce systolic peak pressures of the aortic wall. Although there is only evidence concerning patients with Marfan's syndrome, we also recommend beta-blockers and ACE inhibitors for BAV patients who suffer from comparable risk for rupture or dissection.

### 6.1. Surgical Technique

In consensus with international centers of excellence in aortic surgery we believe that in selected cases of isolated ascending aortic dilation—without involvement of the sinus of valsava—supracommissural ascending aortic replacement is justified in most BAV patients (Sievers type 0 and 1; 95%) [31, 55]. 

Root repair (David operation) or replacement (i.e., with beginning stenosis, Bentall operation) is indicated in BAV patients with ectasia/dilation involving the aortic root (Sievers type 2; 5%).

Valve sparing root repair is—even in bicuspid patients—the preferred approach by specialized centers of excellence in this particular field of reconstructive aortic root surgery [56]. Major limitations obviously apply if anatomy of the bicuspid valve is unsuitable, Aicher et al.:* “Recurrence and progression of regurgitation, however, may occur, depending primarily on anatomic features of the valve”* [56]. While preserving the tricuspid valve aims for regeneration of *natural hemodynamics of the native valve*, this aim is not always desirable in patients with BAV with naturally altered, distorted flow architecture in the ascending aorta/root. 

The Bentall operation in patients with BAV—especially if the sinus of valsalva is dilated—might therefore offer superior durability, in particular if a perfect valve sparing procedure is not achievable [18, 55–57].

Some authors recommend aortic wrapping in the presence of aortic ectasia. In our opinion, this approach cannot be recommended since potentially pathological tissue will remain in place baring a constant risk of aortic rupture.

Based on the fact that the proximal aortic arch rarely is involved or dilated we and other reference centers recommend hemiarch replacement only in very selected cases [18, 58].

With on ongoing controversy regarding whether or not the pulmonary trunk is affected by pathology of common embryologic origin, a prudent consideration when opting for the Ross procedure in patients with bicuspid aortic valve and ascending/root dilation is warranted.

### 6.2. Postoperative Aortic Followup

Aortic disease is cured after proximal aortic repair since downstream dilation, ectasia, or aneurysm formation of the thoracic and thoracoabdominal aorta is extremely rare in patients with BAV [18, 59]. We therefore recommend downstream aortic surveillance in regular intervals only in high-risk patients with familial predisposition or 1st degree relatives with acute aortic dissection.
